# Synthesis, Characterization, and Biological Properties of Iron Oxide Nanoparticles Synthesized from *Apis mellifera* Honey

**DOI:** 10.3390/molecules28186504

**Published:** 2023-09-07

**Authors:** Hamna Shahid, Aqeel Ahmed Shah, Syed Nizam Uddin Shah Bukhari, Anjum Zehra Naqvi, Iqra Arooj, Mehvish Javeed, Muhammad Aslam, Ali Dad Chandio, Muhammad Farooq, Sadaf Jamal Gilani, May Nasser Bin Jumah

**Affiliations:** 1 Department of Microbiology & Molecular Genetics, Faculty of Life Sciences, The Women University, Multan 66000, Pakistan; hamnashahid185@gmail.com (H.S.); mehvish.mmg23@wum.edu.pk (M.J.); 2 Wet Chemistry Laboratory, Department of Metallurgical Engineering, NED University of Engineering and Technology, University Road, Karachi 75270, Pakistan; aqeelshah@cloud.neduet.edu.pk (A.A.S.); alidad@neduet.edu.pk (A.D.C.); 3 Department of Basic Science and Humanities, Dawood University of Engineering and Technology, Karachi 74800, Pakistan; nizamuddin@duet.edu.pk; 4 Department of Microbiology, University of Karachi, Karachi 75270, Pakistan; aznaqvi@uok.edu.pk; 5 Institute of Physics and Technology, Ural Federal University, Mira Str. 19, 620002 Yekaterinburg, Russia; aslam@urfu.ru; 6 Pakistan Council of Scientific and Industrial Research (PCSIR), PCSIR Head Office, 01-Constitution Avenue, Sector G-5/2, Islamabad 44000, Pakistan; mfaruq752@gmail.com; 7 Department of Basic Health Sciences, Foundation Year, Princess Nourah bint Abdulrahman University, Riyadh 11671, Saudi Arabia; sjglani@pnu.edu.sa; 8 Biology Department, College of Science, Princess Nourah bint Abdulrahman University, Riyadh 11671, Saudi Arabia; mnbinjumah@pnu.edu.sa; 9 Environment and Biomaterial Unit, Health Sciences Research Center, Princess Nourah bint Abdulrahman University, Riyadh 11671, Saudi Arabia; 10 Saudi Society for Applied Science, Princess Nourah bint Abdulrahman University, Riyadh 11671, Saudi Arabia

**Keywords:** green synthesis, honey mediation, Fe_2_O_3_-NPs, antibacterial activity

## Abstract

Green approaches for nanoparticle synthesis have emerged as biocompatible, economical, and environment-friendly alternatives to counteract the menace of microbial drug resistance. Recently, the utilization of honey as a green source to synthesize Fe_2_O_3_-NPs has been introduced, but its antibacterial activity against one of the opportunistic MDR pathogens, *Klebsiella pneumoniae*, has not been explored. Therefore, this study employed *Apis mellifera* honey as a reducing and capping agent for the synthesis of iron oxide nanoparticles (Fe_2_O_3_-NPs). Subsequent to the characterization of nanoparticles, their antibacterial, antioxidant, and anti-inflammatory properties were appraised. In UV-Vis spectroscopic analysis, the absorption band ascribed to the SPR peak was observed at 350 nm. XRD analysis confirmed the crystalline nature of Fe_2_O_3_-NPs, and the crystal size was deduced to be 36.2 nm. Elemental analysis by EDX validated the presence of iron coupled with oxygen in the nanoparticle composition. In ICP-MS, the highest concentration was of iron (87.15 ppm), followed by sodium (1.49 ppm) and other trace elements (<1 ppm). VSM analysis revealed weak magnetic properties of Fe_2_O_3_-NPs. Morphological properties of Fe_2_O_3_-NPs revealed by SEM demonstrated that their average size range was 100–150 nm with a non-uniform spherical shape. The antibacterial activity of Fe_2_O_3_-NPs was ascertained against 30 clinical isolates of *Klebsiella pneumoniae*, with the largest inhibition zone recorded being 10 mm. The MIC value for Fe_2_O_3_-NPs was 30 µg/mL. However, when mingled with three selected antibiotics, Fe_2_O_3_-NPs did not affect any antibacterial activity. Momentous antioxidant (IC_50_ = 22 µg/mL) and anti-inflammatory (IC_50_ = 70 µg/mL) activities of Fe_2_O_3_-NPs were discerned in comparison with the standard at various concentrations. Consequently, honey-mediated Fe_2_O_3_-NP synthesis may serve as a substitute for orthodox antimicrobial drugs and may be explored for prospective biomedical applications.

## 1. Introduction

Nanotechnology has attracted immense attention during the last two decades owing to its diverse applications in various fields of science. The versatility of nanoparticles is particularly evident due to their small size, which is usually less than 100 nm [1,2]. A reduction in size determines several features of nanoparticles, including enhanced surface area and improved magnetic and electrochemical properties [3]. Organic product synthesis with nanotechnology is an evolving area because nanotechnology offers numerous benefits in the natural delivery of drugs to heal chronic illnesses [4]. Metal oxide nanoparticles are being studied frequently nowadays due to their unique features [5]. Magnetic nanoparticles of metals, particularly iron oxide nanoparticles (Fe_2_O_3_-NPs), with validated biological strength and biocompatibility, are the focus of applications, including drug delivery, magnetic resonance imaging, and bioremediation [6,7,8]. Hematite (Fe_2_O_3_), magnetite (Fe_3_O_4_), and limonite (Fe_2_O_3_ H_2_O) are the most common forms of iron oxides [9,10]. Fe_2_O_3_-NPs can be synthesized by using chemical methods such as microemulsions, co-precipitation of Fe^2+^ ions, the sol–gel method, sonochemistry, the colloidal method, non-aqueous routes, the pyrolysis reaction, and emulsion techniques [11,12,13,14]. These methods produce harmful chemicals. So, there has been an expanding incentive to use microorganisms and green approaches in Fe_2_O_3_-NP synthesis [15].

A recent study reported the successful synthesis of Fe_2_O_3_-NPs by using aqueous extracts of *Azadirachta indica* [16]. Similarly, other recent studies have reported the synthesis of Fe_2_O_3_-NPs from aqueous extracts of *Ficus carica* and *Hylocereus undantus* [3,16,17]. Various sugars, terpenoids, polyphenols, alkaloids, phenolic acids, and proteins are commonly found in natural extracts and account for their reducing and capping abilities, resulting in stable maintenance of the nanoparticle structure. It has been proven that functional groups such as C-O-C, C-O, C=C, and C=O present in these compounds can markedly assist nanoparticle synthesis [18,19]. *Apis mellifera* honey has been used since ancient times for its therapeutic value [20]. Honey is a natural organic source of nutrition that contains a large amount of glucose and fructose (75%) that act as reducing and stabilizing agents in nanoparticle synthesis [21]. Honey-mediated nanoparticle synthesis does not produce any hazardous by-products that are harmful to human health when used in medical applications. Additionally, honey-mediated green synthesis is a biocompatible, quick, and easy approach that may be utilized to produce a wide range of beneficial end-products in diverse applications [22]. In the past few years, several studies have surfaced on the successful synthesis of biogenic Fe_2_O_3_-NPs [4].

Recently, researchers reported significant in vitro antibacterial potential of honey-loaded Fe_2_O_3_ nanoparticles against *P. aeruginosa*, *B. subtilis*, *S. aureus*, *E. coli*, *Penicillium* spp., and *Aspergillus* spp. [23]. Another study reported the successful synthesis of Fe_2_O_3_-NPs from *Oscillatoria limnetica*. Researchers reported significant antibacterial activities of Fe_2_O_3_-NPs against MDR *E. coli*, *S. aureus*, and *B. subtilis* [24]. Likewise, Fe_2_O_3_-NPs were produced effectively by utilizing gum extract from *Bombax malabaricum*. It was discovered that the bioactive compounds and phytochemicals included in the gum extract of *B. malabaricum* were responsible for the creation of Fe_2_O_3_-NPs. In that study, the antibacterial activity of synthesized NPs was studied against *S. aureus*, *B. halodurans*, and *M. luteus* with significant results [25]. The antibacterial potential of Fe_2_O_3_-NPs is of particular relevance in present times because the spread of antibiotic-resistant species has become a key problem for global health [10]. Fe_2_O_3_-NPs have been proven to be effective against a variety of infectious microorganisms due to their ability to generate highly reactive oxygen species [26]. It is hypothesized that the antimicrobial effect of Fe_2_O_3_-NPs is mainly related to their morphological and physiological characteristics [27].

*Klebsiella pneumoniae* is a proactive pathogen that causes an array of diseases in immunodeficient individuals, such as diabetic patients, neonates, and cancer patients [28]. Multidrug resistance and the emergence of hypervirulent strains of *K. pneumoniae* have been reported and are responsible for a 42% mortality rate in patients and healthy individuals [29]. However, to the best of our knowledge, the in vitro antibacterial potential of Fe_2_O_3_-NPs, specifically against the clinical isolates of *K. pneumoniae,* has not been assessed before. Hence, in this research, pure honey was utilized as a reducing and stabilizing agent to produce Fe_2_O_3_-NPs by using iron chloride hexahydrate (FeCl_3_•6H_2_O) and sodium hydroxide. Further, the characterization of biogenic nanoparticles was performed using numerous techniques, and their antibacterial, antioxidant, and anti-inflammatory potentials were evaluated.

## 2. Results

### 2.1. Visual Observation and UV-Vis Spectroscopy

**Figure 1:**
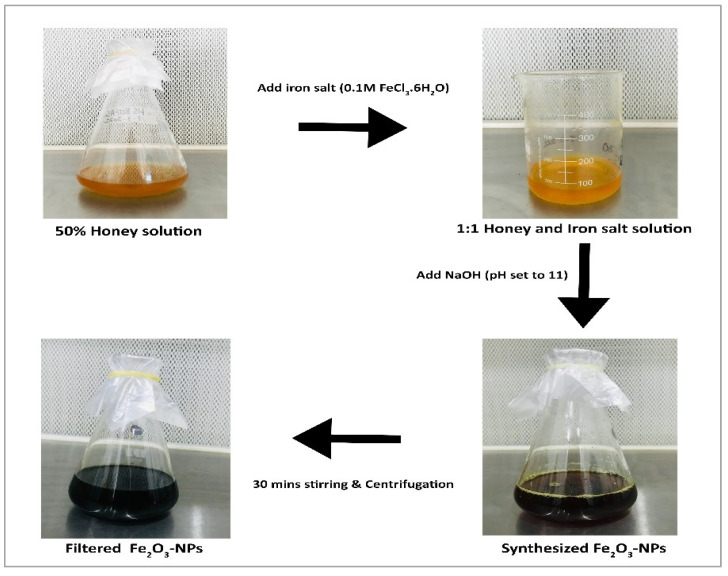
Visual observation of color change from yellow to intense black during synthesis of Fe_2_O_3_-NPs.

**Figure 2:**
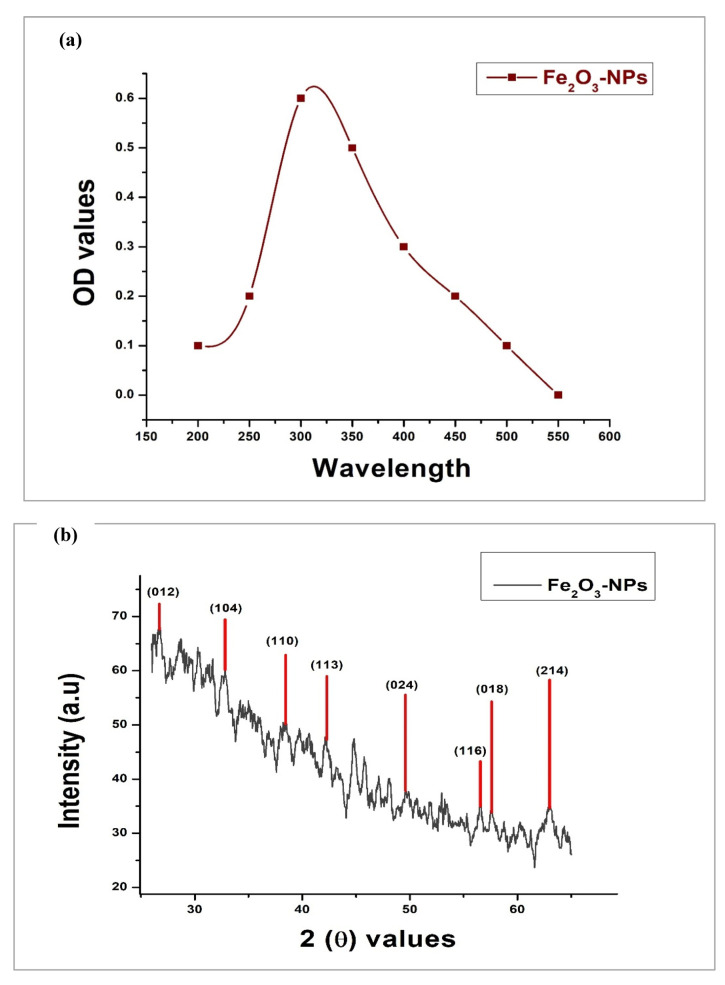

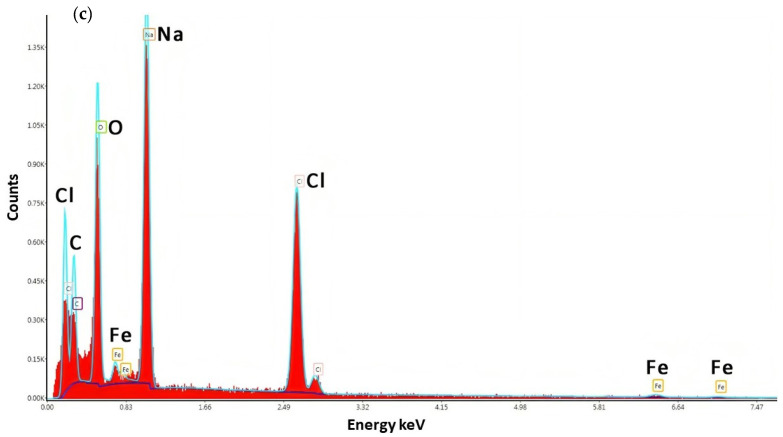
Results representing the characterization of biogenic Fe_2_O_3_-NPs. (**a**) UV–visible graph (a sharp peak at 350 nm is attributed to Fe_2_O_3_-NPs); (**b**) XRD graph (highlighted peaks are attributed to crystalline nature of Fe_2_O_3_-NPs); (**c**) EDX graph (clear Fe and O peaks).

*A. mellifera* honey was recognized as a good source for the synthesis of Fe_2_O_3_-NPs. In the present study, the production of Fe_2_O_3_-NPs started when NaOH was gradually added with stirring to a primary solution containing iron salt and sodium hydroxide. The development of an intense black color provided a visual indication of the presence of nanoparticles in the solution (Figure 1). Primary confirmation was carried out with the help of UV-Vis spectroscopic analysis, which showed a sharp peak at 350 nm, confirming the surface plasmon resonance of the synthesized Fe_2_O_3_-NPs (Figure 2a).

### 2.2. Characterization by XRD, EDX, ICP-MS, VSM, and SEM

In order to determine the single phase and crystalline nature of nanoparticles, XRD analysis was carried out. As represented in Figure 2b, intense peaks at 2θ values of 24.68°, 33.88°, 38.44°, 41.36°, 49.8°, 56.64°, 57.48°, and 62.88° corresponding to index values of (012), (104), (110), (113), (024), (116), (018), and (214) were observed for Fe_2_O_3_-NPs. These values were contrasted with those of the Joint Committee on Powder Diffraction Standards (JCPDS No. 24-0072) for Fe_2_O_3_-NPs. These crystal planes clearly represent the crystalline nature of Fe_2_O_3_-NPs in the sample, and the average crystal size was measured as 36.2 nm by using the Debye–Scherer equation (D = *K ƛ/β*1/2*CosƟ*) with an average lattice strain of 0.00285. These crystal planes indicated that synthesized Fe_2_O_3_-NPs may be in a rhombohedral crystal phase with lattice constants of a = b = 5.0346 Å and c = 13.7473 Å. These lattice constant parameters were matched with the Crystallography Open Database (COD No. 9015964).

The EDX analysis shown in Figure 2c revealed the elemental composition of the synthesized Fe_2_O_3_-NP solution. K-α peaks between 0.0 and 0.83 keV confirmed that Fe and O are present in the synthesized nanoparticles. The abundance of oxygen demonstrated that the nanoparticles are in iron oxide form. Peaks of C and O atoms confirmed the contribution of honey in the synthesis of nanoparticles. The presence of Na and Cl atoms was also detected as impurities in the solution. In the present study, the highest proportion of Fe elements was found in the EDX spectra, indicating that the main component was Fe_2_O_3_-NPs (Table 1).

ICP-MS analysis was performed to determine the trace element concentration in the synthesized Fe_2_O_3_-NPs. The concentration of each trace element was determined in ppm (parts per million) at different wavelengths. According to the results, Fe was found to be at its highest concentration of 87.15 ppm at a wavelength of 238.204 nm with an intensity of 762,552.31 counts per second (c/s), while a 1.49 ppm concentration was measured for Na, which was found at 589.590 nm with an intensity of 508.64 c/s (Table 2).

**Figure 3:**
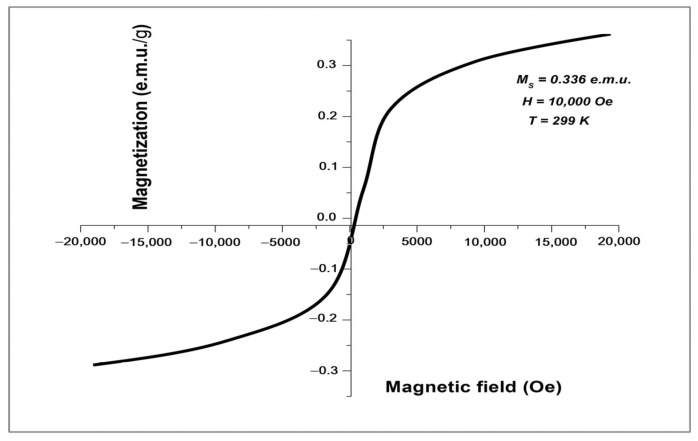
Magnetic behavior of Fe_2_O_3_-NPs as measured by vibrating sample magnetometry.

**Figure 4:**
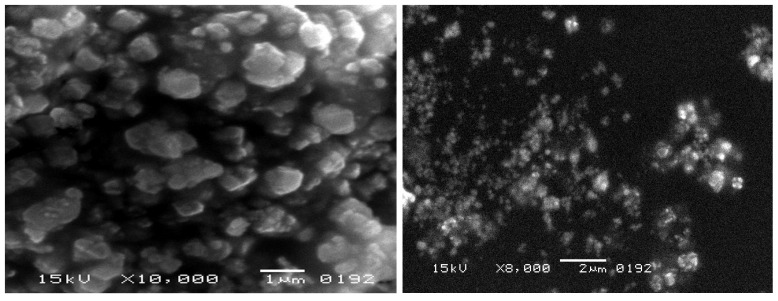
SEM images of Fe_2_O_3_-NPs at magnification up to 10,000x.

Figure 3 shows the magnetic hysteresis loop (MH-loop) for synthesized hematite Fe_2_O_3_-NPs at 299 K. The curve revealed the weak ferromagnetic behavior of the synthesized Fe_2_O_3_-NPs with a saturation magnetization (M_S_) value of 0.336 e.m.u./g at 1 Tesla (10,000 Oe). The morphological (size and shape) properties of the produced Fe_2_O_3_-NPs, as determined by SEM, are depicted in Figure 4. These images confirmed the development of nanostructures, which were well distributed in the solution. A continuous variation was observed in the shape and size of the produced Fe_2_O_3_-NPs. At 19,000x magnification, the size distribution of Fe_2_O_3_-NPs was estimated to be in the range of 100–200 nm with a non-uniform spherical shape.

### 2.3. Antibacterial Studies

**Figure 5:**
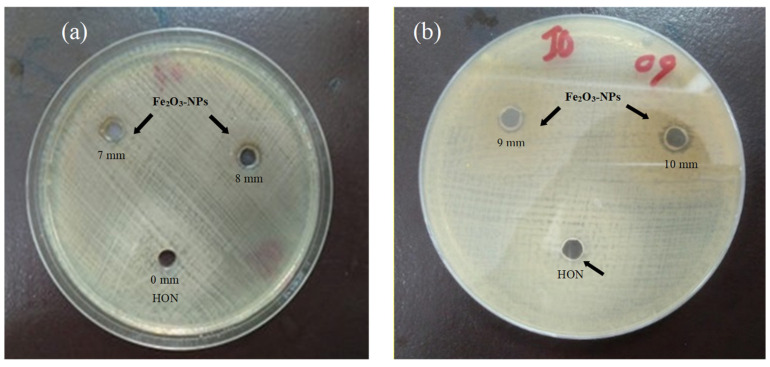
Antibacterial potential of Fe_2_O_3_-NPs. (**a**,**b**) MH agar plates display the antibacterial property of Fe_2_O_3_-NPs against *K. pneumoniae*; (**b**) arrow is pointing towards the minimal antibacterial effect of honey in comparison with Fe_2_O_3_-NPs against *K. pneumoniae*.

In the present research, the antibacterial activity of newly synthesized Fe_2_O_3_-NPs was studied in detail against one of the notorious opportunistic Gram-negative bacteria, *K. pneumoniae*. A 30 µg/mL concentration of Fe_2_O_3_-NPs revealed the most significant in vitro antibacterial property against all isolates of *K. pneumoniae*, as demonstrated by the measured zone diameters (Figure 5a). However, the honey solution did not show any bactericidal effect when used alone, as no zones of inhibition could be observed (Figure 5b). Among all isolates, the largest inhibition zone of 10 mm was observed against HS-K4, HS-K-5, HS-K9, and HS-K-15. On the other hand, the lowest inhibition zone of 5 mm was recorded against HS-K-17 and HS-K-19. Intermediate values of 6 mm to 9 mm were observed against the other 24 isolates of *K. pneumoniae*.

The MIC value for synthesized Fe_2_O_3_-NPs against *K. pneumoniae* was interpreted as 30 µg/mL based on ELISA reader absorbance values. After incubation, the in vitro efficacy of antibiotics was examined against clinical strains of *K. pneumoniae*. The antibacterial activity of nanoparticles was also measured in combination with three selected antibiotics and compared with the individual antibacterial potential of nanoparticles and antibiotics. The results were equated to CLSI guidelines and interpreted as follows: all strains of *K. pneumoniae* showed an intermediate resistant (I) pattern against CN-10 and FEP-30, while all strains showed sensitivity (S) to CIP-5. Surprisingly, when antibiotics and nanoparticles were used in combination, no zones of inhibition could be seen at all.

### 2.4. Antioxidant Potential

**Figure 6:**
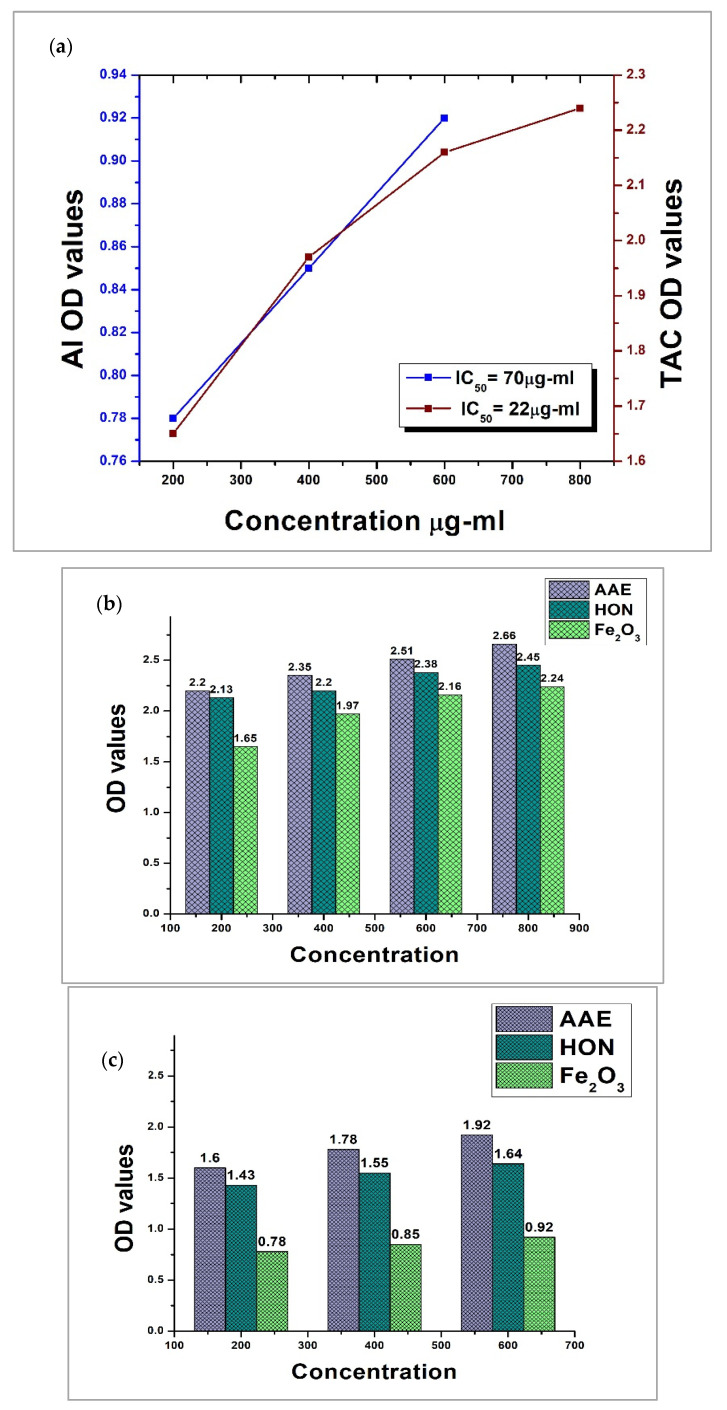
Antioxidant and anti-inflammatory potential of Fe_2_O_3_-NPs. (**a**) IC_50_ values for total antioxidant capacity (TAC) and anti-inflammatory (AI) activity of Fe_2_O_3_-NPs in terms of ascorbic acid (AAE) and honey (HON); (**b**) TAC values for different concentrations of Fe_2_O_3_-NPs in terms of AAE and HON; (**c**) AI values for different concentrations of Fe_2_O_3_-NPs in terms of AAE and HON.

The antioxidant potential of Fe_2_O_3_-NPs was found to be superior to that of the standard. At concentrations of 200, 400, 600, and 800 µg/mL, absorbance values of 1.65, 1.97, 2.16, and 2.24 were measured for Fe_2_O_3_-NPs, respectively. At the same concentrations of AAE, which were included as a standard, absorbance values of 2.2, 2.35, 2.51, and 2.66 were recorded, respectively. The absorption readings for HON at the concentrations of 200 µg/mL, 400 µg/mL, 600 µg/mL, and 800 µg/mL were 2.13, 2.2, 2.38, and 2.45, respectively. Figure 6a represents the IC_50_ value in terms of total antioxidant capacity (TAC), and it was calculated to be 22 µg/mL for Fe_2_O_3_-NPs. Furthermore, it was interpreted from the graph included in Figure 6b that the antioxidant capacity of nanoparticles correspondingly increased with an increase in their concentration in the sample.

### 2.5. Anti-Inflammatory Potential

Honey-mediated Fe_2_O_3_-NPs manifested considerably greater anti-inflammatory potential as compared to the standard. Absorbance values of 0.78, 0.85, and 0.92 were measured for the synthesized nanoparticles at different concentrations of 200, 400, and 600 µg/mL, respectively. On the contrary, at the same concentrations, AAE was demonstrated to possess absorbance values of 1.6, 1.78, and 1.92, respectively, whereas the spectrophotometer readings for HON at concentrations of 200 µg/mL, 400 µg/mL, and 600 µg/mL were 1.43, 1.55, and 1.64, respectively. Figure 6a illustrates the IC_50_ value for Fe_2_O_3_-NPs in terms of anti-inflammatory potential (AI), and it was calculated to be 70 µg/mL. From the graph shown in Figure 6c, it was deduced that anti-inflammatory capacity increased as the concentration of Fe_2_O_3_-NPs increased in the solution.

## 3. Discussion

Fabrication of Fe_2_O_3_-NPs utilizing the capping and stabilizing agents in biological resources is a biocompatible, eco-friendly, and nontoxic approach with widespread biomedical applications. Recently, these nanoparticles have drawn considerable attention owing to the magnetic properties and flexible surface chemistry of iron oxide [30]. The utilization of honey as a single antecedent in the production of Fe_2_O_3_-NPs produced results that are equivalent to those achieved when using only glucose or fructose. Since honey exhibits an analogous pattern with respect to the size of particle decline during Fe_2_O_3_-NP creation [31], in this study, *Apis mellifera* honey-loaded Fe_2_O_3_-NPs were synthesized and evaluated for their potential biological properties. During synthesis, the appearance of an intense black color provided a visual indication of the successful synthesis of nanostructures. Previous studies have also reported the formation of an intense black color toward the end of the synthesis procedure for Fe_2_O_3_-NPs [7,15,26].

In the present study, Fe_2_O_3_-NPs were characterized in more detail using UV-Vis analysis, which can be utilized to learn essential details about Fe_2_O_3_-NPs’ sturdiness, size, and shape [32]. A clear and intense peak was observed at 350 nm, which corresponded to the presence of Fe_2_O_3_-NPs in the sample. Similar results were described in a previous study that reported the green synthesis of Fe_2_O_3_-NPs [4]. The crystalline structure of biogenic Fe_2_O_3_-NPs was evaluated using XRD analysis. Sharp diffraction peaks were observed at various index values ((012), (104), (110), (113), (024), (116), (018), and (214)), which revealed that the synthesized nanoparticles possessed extremely fine nature, a good crystalline rhombohedral structure, and a calculated size of 36.2 nm (a = b = 5.0346 Å, c = 13.7473 Å). This was in line with previous observations regarding nanoparticle crystal size [33]. Previous studies reported the similar crystal planes of (012), (104), (110), (113), (024), (116), (018), and (214) at similar 2θ values for Fe_2_O_3_-NPs that can be correlated with the current study [26,34].

EDX analysis was used to determine the elemental content of the sample. Iron typically exhibits an intense peak at 0.7–7 keV [35]. The EDX graph clearly depicts the presence of one K-α peak before 0.8 keV, which can be attributed to iron atoms, and two K-α peaks between 0.0 keV and 0.83 keV, which can be ascribed to carbon and oxygen atoms. The presence of carbon and oxygen, together with iron, confirmed the involvement of honey in the synthesis of nanoparticle structures. This led to the production of quantitative as well as qualitative evaluations of the iron components that contributed to the generation of Fe_2_O_3_-NPs [36]. Cl and Na atoms were also found as residual impurities while synthesizing nanoparticles from ferric chloride, and this has been documented in previous studies [37]. Several earlier studies that performed XRD and EDX analyses of Fe_2_O_3_-NPs synthesized from different natural sources reported similar observations [38,39]. Furthermore, the ICP-MS technique was used to measure the exact concentration of elements (Fe and Na) in synthesized nanoparticles. The measured concentrations of Fe and Na were 87.15 ppm (238.204 nm) and 1.49 ppm (589.590 nm), respectively. These results confirmed the presence of Fe atoms, which dominated others in synthesized nanoparticles, pointing towards the highest proportion of Fe_2_O_3_-NPs in the sample. Another study reported a concentration of ultra-filtered Fe_2_O_3_-NPs in suspension, which was in line with our observations [40].

The MH-loop produced by VSM analysis confirmed that the synthesized Fe_2_O_3_-NPs had weak ferromagnetic properties. The M_S_ value of 0.336 e.m.u. can be correlated to Fe_2_O_3_-NPs with bulk hematite, as reported in an earlier study [41]. Some previous studies reported superparamagnetic properties of small-sized (<90 nm) hematite Fe_2_O_3_-NPs with calculated M_S_ values of 8.5 e.m.u./g and 0.96 e.m.u./g at 300 K [42,43]. A previous study reported the anti-ferromagnetic (non-saturating magnetization) properties of hematite Fe_2_O_3_-NPs even at a temperature of 1000 K [44]. The SEM images with a magnification of up to 20,000x revealed that the nanoparticle size distribution was in the range of 100 nm and 200 nm. Recent studies have also demonstrated that the size of iron oxide-based nanoparticles is between 10 nm and 100 nm [45,46]. In the present study, the shape of Fe_2_O_3_-NPs was estimated to be irregularly spherical. A previous study also reported a cavity-like shape with a rough surface of Fe_2_O_3_-NPs [47]. The SEM images of the present study revealed that the precursor (honey) was stabilizing the surface of nanoparticles by selectively slowing their growth rate and stopping particle aggregation. This result can be correlated with a previous similar study [23]. Another study demonstrated that the nature of Fe_2_O_3_-NPs was not uniform and that they were present mostly in the form of large, agglomerated groups. These clusters were linked to the low capping ability of the plant source and the magnetic properties of Fe_2_O_3_-NPs [26].

The antimicrobial activity of honey-mediated Fe_2_O_3_-NPs was evaluated against clinical strains of *K. pneumoniae*, and significant antibacterial activity was observed against all strains of *K. pneumoniae*. The estimated MIC value for the formulated nanoparticles was 30 µg/mL. Significant antibacterial activity of Fe_2_O_3_-NPs synthesized from different organic extracts has been reported previously against various groups of bacteria, including *Pseudomonas* spp., *E. coli*, *S. aureus*, and *B. cereus*, among others [47,48]. A recent study also reported a 10 mm inhibition zone as the highest for Fe_2_O_3_-NPs against *S. aureus* [23]. In line with these findings, the authors noticed that if the concentration of nanoparticles in the solution increased, the antibacterial properties improved. Many studies have been conducted to elucidate the mechanisms, and it has been determined that the antimicrobial activity of nanoparticles is largely dependent on capping agents [49,50]. Several scientists have also found that relatively smaller nanoparticles display better antibacterial potential [51,52]. Although it is still unclear how exactly metal or metal oxide NPs work to kill bacteria, Fe_2_O_3_-NPs work by blocking cellular proteins and enzymes from replicating. According to much research, nanoparticles damage the integrity of cells by penetrating both the cell wall and the membrane, as well as by triggering the death of cells by damaging nucleic acids and proteins [25].

In the present study, no synergistic potential of the synthesized nanoparticles with antibiotics was observed because no zones of inhibition were recorded on the MH agar plates subsequent to the incubation time period. On the contrary, Abo-Shama et al. (2020) and some others have reported good synergistic activity between various metallic nanomaterials and different commercially available antibiotics [53]. In our study, the lack of synergism could be due to the high concentration of honey in the solution, which blocked the diffusion of antibiotics into the agar, or poor chemical compatibility between fabricated nanoparticles and antibiotics. The second hypothesis seems closer to reality, as the antibacterial effect of nanoparticles was missing in the presence of antibiotics. Hence, it can be safely concluded that honey-mediated Fe_2_O_3_-NPs and antibiotics suppressed each other’s antibacterial effects.

The antioxidant potential of different aqueous samples of formulated nanoparticles was measured using the TAC method. The results illustrated the significant antioxidant capacity of all nanoparticle concentrations (200 µg/mL =1.65 OD, 400 µg/mL = 1.97 OD, 600 µg/mL = 2.16 OD, and 800 µg/mL = 2.24 OD), which was approximately equal to that of HON and less than that of standard AAE. After incubation, a very dark blue hue was noticed in the test tubes, which was similar to the control. The IC_50_ value for newly synthesized nanoparticles was estimated at 22 µg/mL in terms of TAC. In the present study, a 50% honey solution was used to produce nanoparticles, and due to the high concentration of honey in the final solution, a very strong antioxidant potential was seen with a very low IC_50_ value. Recently, the good antioxidant potential of nanoparticles based on iron oxide resulting from distinctive biological resources has been observed with significant IC_50_ values (20.456 µg/mL, 58.85 mg/mL, 45.4 mg/mL, 33 mg/mL) [54,55,56].

The anti-inflammatory activity of our synthesized nanoparticles was determined in terms of BSA using the protein inhibition method. Various aqueous concentrations of newly synthesized Fe_2_O_3_-NPs revealed better anti-inflammatory activities (200 µg/mL = 0.78 OD, 400 µg/mL = 0.85 OD, 600 µg/mL = 0.92 OD) in comparison with AAE or HON, and their IC_50_ value was measured at 70 µg/mL. A recent study reported significant anti-inflammatory activity of nano-ointment that was fabricated with iron oxide and zinc oxide nanoparticles [57]. Another study reported the significant percentage inhibition of Fe_2_O_3_-NPs at different concentrations (100 = 25.68%, 200 = 45.43, 300 = 60.59%, 400 = 82.49%, 500 = 92.59%, IC_50_ value = 234.45 µg/mL). The anti-inflammatory activity of these Fe_2_O_3_-NPs revealed their protective nature. It is a common occurrence for secondary or tertiary protein structures to disintegrate, leading to a disruption of biological function [2]. Therefore, it can be stated that honey-mediated synthesis of Fe_2_O_3_-NPs leads to reliable antibacterial, antioxidant, and anti-inflammatory effects, which can be pursued in future studies.

## 4. Materials and Methods

### 4.1. Honey-Mediated Synthesis of Fe_2_O_3_-NPs

Organic honey of *Apis mellifera* (honeybee) was bought from the local market in Multan, Pakistan. For green synthesis, 50% honey solution and 0.1 M FeCl_3_•6H_2_O (98% pure) (1:1) were added to a flask. To this solution, NaOH (99% pure) was added dropwise to set the pH of the solution to 11. The final solution was stirred vigorously for 30 min on a magnetic stirrer until an intense black color was observed. This was an indication of the formation of Fe_2_O_3_-NPs. Centrifugation was performed for 40 min at 8000 r.p.m. to separate nanoparticles from other particles in the solution, and then the pellet was washed with water and ethanol three times. An ultra-fine powder of nanoparticles was obtained by keeping the solution in a hot air oven at 80 °C for 6–7 h [26].

### 4.2. Characterization of Honey-Mediated Fe_2_O_3_-NPs

The bioactive iron oxide nanomaterials were characterized using a variety of analytical techniques, including UV-Vis spectroscopy, X-ray diffraction analysis (XRD), energy-dispersive X-ray analysis (EDX), inductively coupled plasma mass spectrometry (ICP-MS), and scanning electron microscopy (SEM). For primary confirmation, UV-Vis spectrophotometry was performed to determine the spectral frequency of Fe_2_O_3_-NPs in the range of 200–800 nm. Two cleaned cuvettes made of quartz were used for this; one contained water, and the other contained approximately 3 mL of Fe_2_O_3_-NPs. The pure samples were diluted up to 5 times because using undiluted solutions produced noisy outcomes. After that, the UV–visible range was adjusted between 200 and 800 nm, and peaks were recorded. A BRUKER D8 Discover diffractometer with a 50–500 µm X-ray beam was utilized to study the crystal planes of Fe_2_O_3_-NPs. This was accomplished by projecting an X-ray beam onto a powdered specimen from the radiation source, which caused reflections to create various diffraction configurations. The precise specimen’s crystallographic nature was revealed by these diffraction patterns [58].

The involvement of different elements in the synthesis of Fe_2_O_3_-NPs was determined by using the EDAX Team at 12.5 kV, 3.84 A, 500×, and 129 eV. For EDX analysis, an electron beam that generated distinct X-rays in the elements of the sample was employed. The detector detected every wavelength of X-rays that the sample emitted. The individual element (the source of the unique X-ray) found in a sample is represented by each X-ray with a wavelength that varies [59]. ICP-MS is an analytical technique that is usually employed to analyze trace elements present in thermally digested biological samples [60]. Before ICP-MS analysis, the sample was thermally digested by aqua regia (1:3 HCl: HNO_3_) and diluted. For this, 3 mL HNO_3_, 1 mL HCl, and 0.5 g Fe_2_O_3_-NPs were added to a clean test tube. The solution was heated above 100 °C on a hotplate until the visible mass disappeared (transparent yellow color) from the test tube. The resultant solution was filtered, and 9 mL of deionized water was added to 1 mL of the filtered solution. After that, the determination of trace elements in the digested sample was performed using the ICP-MS Shimadzu model (MY-17320003) at different wavelengths and specific conditions: RF power (1.2), viewing height (8 mm), nebulizer flow (0.7), plasma flow (12), aux flow (1), and make-up flow (0).

The moment of magnetization of prepared Fe_2_O_3_-NPs was observed using a vibrating sample magnetometer (VSM) at a temperature of 299.0 K with an applied magnetic field of up to 20,000 Oe. The physical properties of honey-mediated Fe_2_O_3_-NPs were analyzed by scanning electron microscopy (SEM). Real-time pictures of the samples, clarity at the nanometer level of 20 to 100 nm, and a broad traversing zone of nanoparticles are all provided by this methodology [61]. The SEM instrument JSM-6380 with a potential energy of 15 KV and magnification up to 20,000x was used to examine morphological properties (size and shape) of the synthesized Fe_2_O_3_-NPs.

### 4.3. Estimation of Antibacterial Potential and MIC

Between October 2021 and December 2021, 30 clinical strains of *K. pneumoniae* were collected from the Pathology laboratory of Nishtar Hospital, Multan, and were named HS-K-1 to HS-K-30. The antibacterial activity of Fe_2_O_3_-NPs was determined against selected clinical strains of *K. pneumoniae* using the agar well diffusion method. Mueller–Hinton (MH) agar plates were aseptically prepared, and each plate was swabbed with a 0.5 McFarland standard bacterial culture. After drying for 15 min, 3 wells of equal width were generated on each plate using a pipette. The first two wells were loaded with 30 µL of Fe_2_O_3_-NPs, and the last well was loaded with a diluted honey solution. All plates were incubated at 37 °C overnight. The next day, inhibition zones were measured for each strain, and results were recorded in accordance with CLSI guidelines. By using a sterile 96-well plate, the MIC value of Fe_2_O_3_-NPs was determined. Aqueous solutions of nanomaterials (20–50 µg/mL) were prepared in sterile broth. In each of the wells, 180 µL of nanoparticle dilution and 20 µL of 0.5 McFarland standard *K. pneumoniae* culture were mixed. After 20 h of incubation at 37 °C, optical density was recorded in an ELISA plate reader (OD_620_). All the analyses were performed in triplicate.

### 4.4. Synergism with Antibiotics

The Kirby–Bauer disc diffusion method was used to analyze the susceptibility profile of bacteria against three selected antibiotics: ciprofloxacin (CIP-5), gentamicin (CN-10), and cefepime (FEP-30). MH agar plates were prepared, and bacterial lawns were made on agar plates using 0.5 McFarland standard *K. pneumoniae* cultures aseptically. After that, antibiotic discs were impregnated on agar with the help of syringes, and plates were incubated at 37 °C overnight. For determining the synergistic activity of Fe_2_O_3_-NPs with selected antibiotics, a well diffusion procedure was performed with antibiotics soaked in nanoparticle solution in wells. Subsequent to incubation, zones of inhibition were measured and recorded in accordance with CLSI guidelines for 2022.

### 4.5. Evaluation of Antioxidant Potential

The antioxidant capacity of Fe_2_O_3_-NPs was determined using a phosphomolybdenum spectrometric assay. A solution containing 4 mM ammonium molybdate, 0.6 mM sulfuric acid, and 28 mM sodium phosphate was prepared. Different initial concentrations (200–800 µg/mL) of Fe_2_O_3_-NPs were also prepared. Ascorbic acid (AAE) and honey solution (HON) were included as standards. Following preliminary preparations, 1 mL of sample was mixed with 100 µL of each initial concentration in separate test tubes. Test tubes were incubated in a pre-warmed (95 °C) water bath for an hour and a half. Afterward, the test tubes were removed from the water bath and cooled down to room temperature. Finally, the absorbance of each sample was measured at 695 nm. Total antioxidant capacity (TAC) values were calculated by using the following formula:

Total antioxidant capacity (%) = (OD_control_ − OD_sample_)/OD_control_ × 100 (1)

The final results were interpreted as a half-maximal inhibitory concentration (IC_50_) value.

### 4.6. Determination of Anti-Inflammatory Potential

For this experiment, an initial solution of 0.2% (*w*/*v*) bovine serum albumin (BSA) and well as different concentrations of Fe_2_O_3_-NPs (200–800 µg/mL) were prepared. As standards, ascorbic acid (AAE) and honey solution (HON) were used. Then, 5 mL of BSA solution was mixed with 50 µL of each of the different nanoparticle concentrations in separate test tubes, which were then incubated in a pre-warmed (75 °C) water bath for five minutes. Subsequently, test tubes were removed from the water bath and cooled down to room temperature. Each sample’s absorbance was measured at 660 nm in a spectrophotometer. Anti-inflammatory potential values were calculated by using the following formula:

Anti-inflammatory capacity % = (OD_control_ − OD_sample_)/OD_control_ × 100 (2)

The final results were interpreted as a half-maximal inhibitory concentration (IC_50_) value.

## 5. Conclusions

In conclusion, nanotechnology has become a rapidly expanding branch of science in recent years due to widespread applications in the environment, industries, electronics, and medicine. Organic honey was used in the present investigation for the green production of nanoparticles based on iron oxide, representing a safe, sustainable, nontoxic, easily accessible, and economical approach. The phytochemical constituents present on the particle surface lower the chances of clumping, making the Fe_2_O_3_-NPs fabricated in the present study more stable than chemically produced nanoparticles. Additionally, they have the advantage of being simpler to separate as compared to other metal oxide nanoparticles, which call for extremely labor-intensive centrifugation. Furthermore, these nanoparticles exhibited considerable antibacterial, antioxidant, and anti-inflammatory actions against clinically isolated *K. pneumoniae* strains. Together, this makes them candidates for promising antimicrobial agents with prospective biomedical applications.

## Figures and Tables

**Table 1:** The elemental composition of synthesized Fe_2_O_3_-NPs as per the EDX analysis.

Element	Weight %	Atomic %	Net Int.
Fe O	40.73	50.48	363.77
Na Cl	7.58	4.87	40.73

**Table 2:** The trace element composition of synthesized Fe_2_O_3_-NPs as per the ICP-MS analysis.

Trace Elements	Wavelength (nm)	Concentration (ppm)
Fe	238.204	87.15
Na	589.590	1.49
As	188.980	0.02
Cd	214.439	0.09
Co	238.892	0.03
Cr	267.716	0.02
Cu	327.395	−0.03
Mg	279.553	0.02
Mn	257.610	0.02
Ni	231.604	0.07
Pb	220.353	0.06
Zn	213.857	0.02
Se	196.026	0.02

## Data Availability

Data will be available from authors on reasonable request.

## Sample Availability

Not available.
